# Characteristics of Optimal Cancer Referrals Made by Primary Care Clinicians: Scoping Review

**DOI:** 10.1177/10732748251359405

**Published:** 2025-07-28

**Authors:** Olufisayo Olakotan, Judith Yargawa, Julie-Ann Moreland, Claire Friedemann Smith, Brian D. Nicholson, Andrew Millar, Georgia B. Black

**Affiliations:** 1 Wolfson Institute of Population Health, 105714 Queen Mary University of London, London, UK; 2 Department of Radiology, 6397 Oxford University Hospitals NHS Foundation Trust, Oxford, UK; 3 Nuffield Department of Primary Care Health Sciences, Radcliffe Observatory Quarter, University of Oxford, UK; 4 4963 North Middlesex University Hospital NHS Trust, London, UK

**Keywords:** Cancer, referral, healthcare improvement, communication

## Abstract

**Background:**

In England, over 2 million patients are referred each year on urgent pathways to investigate suspected cancer. The content and quality of referrals have often been audited, but there is no consensus on what should be included in a referral to optimise diagnostic outcomes.

**Aim:**

To identify and describe the characteristics of referral letters for suspected cancer from primary to secondary care that may optimise diagnostic outcomes.

**Method:**

The scoping review employed the methodology developed by Arksey and O'Malley in 2005 and further expanded by Levac and Colquhoun 2010. We searched PubMed, Embase, and PsycINFO to identify relevant studies in English Language published between 2000 and 2023. All findings were reported according to PRISMA guidelines for scoping reviews.

**Results:**

Of 3463 identified records, only thirteen met the inclusion criteria, employing qualitative and mixed methods, as well as retrospective audits of referrals. The studies noted that symptom information such as duration, appearance, and descriptive qualities was often missing. There was limited evidence suggesting that the inclusion of clinical examination findings, test information, and the motivation of the referring clinician were beneficial. Evidence relating to the benefits of guidelines and template referral forms was mixed. There was a paucity of research linking referral content to patient diagnostic outcomes.

**Conclusion:**

Despite a small number of studies retrieved, there was broad consensus about the benefit of conveying detailed information in referrals for suspected cancer, particularly with respect to comprehensive symptom description and relevant tests and clinical examinations. Further research linking referral quality to diagnostic outcomes would be beneficial to drive improvement to diagnostic outcomes.

## Plain Language Summary

Every year in England, primary care clinicians urgently refer over 2 million people for cancer tests. However, there is no clear agreement on what information these referrals should include to get the best results for patients. This study looked at research about what makes a good cancer referral letter. Out of more than 3,400 studies that we looked at, only 13 had relevant evidence. Many referral letters lacked key details, such as how long symptoms had lasted (e.g. feeling tired) or what they looked like (e.g. a rash or a spot). Some evidence suggested that it could be useful to including test results and physical examination findings. Some research also suggested that it was helpful if the doctor wrote down why they were concerned about cancer in the referral letter. However, it was unclear whether using guidelines or templates made a difference. In general, clear and detailed referral letters seem to help, but there is little research proving they lead to better cancer diagnosis. More studies are needed to understand how improving referrals can benefit patients.

## Introduction

Since 1999, the UK has had cancer policies to expedite access to specialists for investigation of a suspected cancer.^1^ The decision to make a suspected cancer referral falls to the primary care clinician who completes a referral form designed to convey all the relevant information to the secondary care team. The receiving team in local secondary care services then timetable patients’ appointments to fit within the required timeframe. Since 2020, the National Health Service (NHS) England Faster Diagnosis Standard has determined that patients urgently referred for a suspected cancer should find out whether they have cancer within 28 days from referral. Similarly managed cancer diagnostic pathways have been implemented in many countries, including Denmark, Norway, and Sweden, with general practitioners making referrals through either site-specific pathways (eg, lung, colorectal) or non-specific symptom pathways.^2-5^ Site-specific systematic reviews present a mixed picture of effectiveness of urgent cancer referral pathways, with improved standardisation compared to non-urgent referrals but limited impact on stage at diagnosis.^6,7^

Cancer referral letters are the conduit to transfer clinical responsibility for urgent cancer investigation between primary and secondary care, transmitting the reason for referral, the required information and the expectations of the referring practitioner (eg, to receive management advice, particular tests or investigations, reassurance for the patient and so on).^8^ A referral letter should result in the patient being seen quickly, by the right specialist and in a patient-centred manner.^9^ Local audit studies have shown that referral letters for suspected cancer can lack adequate information for specialists to make informed decisions regarding prioritization, triage, or the most suitable destination for the referral.^10,11^ Incomplete information can cause errors and inefficiencies, as teams expend resources searching for patient records, having telephone calls with patients and/or carers, and with other clinicians to determine key parts of the patient’s history.^10-12^ Another problem raised by primary care clinicians was the difficulty in accessing the appropriate form for referral to the chosen specialty and hospital.^13^ Interventions such as structured proformas by letter, or electronic referral systems linking the forms to the patient record have been implemented to improve the consistency and quality of cancer referral letters.^9^ Despite the importance of timely referral by GP in the context of suspected cancer, there are no guidelines for what to include in a referral document. This scoping review aimed to examine and summarise the evidence base for optimal cancer referral documents, and to generate characteristics or criteria that could be applied to guidelines, referral proformas or cancer referral audits.

## Methods

This scoping review employs the methodology developed by Arksey and O'Malley in 2005^14^ and further expanded by Levac and Colquhoun 2010.^15^ The widely used method involves 5 key steps: identifying the research question, identifying relevant studies, selecting the studies, charting the data, collating, summarizing, and reporting the results. All findings were reported according to PRISMA guidelines for scoping reviews.

### Research Question

What are the optimal characteristics of primary care referral forms for cancer patients, and how do these affect diagnostic outcomes?

### Identifying Relevant Studies

We searched the databases PubMed, Embase, and PsycINFO to identify relevant studies published between 2000 and 2023. This involved a combination of keywords, Medical Subject Heading (MeSH) terms and search strings that are specific to each database as shown in Online Appendix B. We included terms specific to each database relating to primary care, cancer, and referral. We did not specifically limit the search to urgent referrals. Additionally, reference lists of relevant studies were manually searched.

### Eligibility Criteria

Eligible studies included articles providing empirical evidence (ie, qualitative, quantitative, or mixed methods) on optimal cancer referral forms for adult symptomatic patients across all relevant specialties, including evidence relating to referral criteria, reasons for referral, appropriateness, quality of referral forms, and destination. We included articles from all countries, despite differences in referral systems and structures.

We excluded studies on cancer genetic testing, palliative care referrals, adherence to guidelines, pathways to cancer diagnosis excluding referral content, appropriateness of referrals without any reflection on the referral form/process/software supporting primary care clinicians, and paediatric cancer patients. Additionally, non-research publications such as reports, editorials, and conference abstracts, as well as non-English language articles, were excluded. We uploaded all retrieved articles to Rayyan, where duplicates were removed. Afterwards, one author (OO) independently screened article titles. Three reviewers (GB, JY, EGM) each screened 20% of the abstracts, while OO reviewed all. Conflicting decisions were resolved through discussion, leading to modification of inclusion and exclusion criteria. Subsequently, two reviewers (JY and GB) screened 20% of the full-text articles, while (OO and EGM) reviewed all, resolving disagreements through discussion. For the final selection of full-text articles, three reviewers (OO, JY, GB) met in person to reach consensus.

### Data Charting

A standardized data extraction form was used to extract information such as referral characteristics, outcome measures, key findings, methods used to capture data, study identification, and characteristics from the selected articles. The key findings were coded by OO and GB into broader themes and sub-themes, and articles were assessed for quality using the Mixed Methods Appraisal Tool (MMAT).

### Collating, Summarising and Reporting the Findings

A descriptive summary of study characteristics, such as study designs, number of included studies, and publication years, is included in the review. Subsequently, a narrative synthesis was used to identify key themes, patterns, trends, similarities, and differences across specialties, incorporating quotes to support the synthesis of findings where needed.

## Results

**Figure 1.**
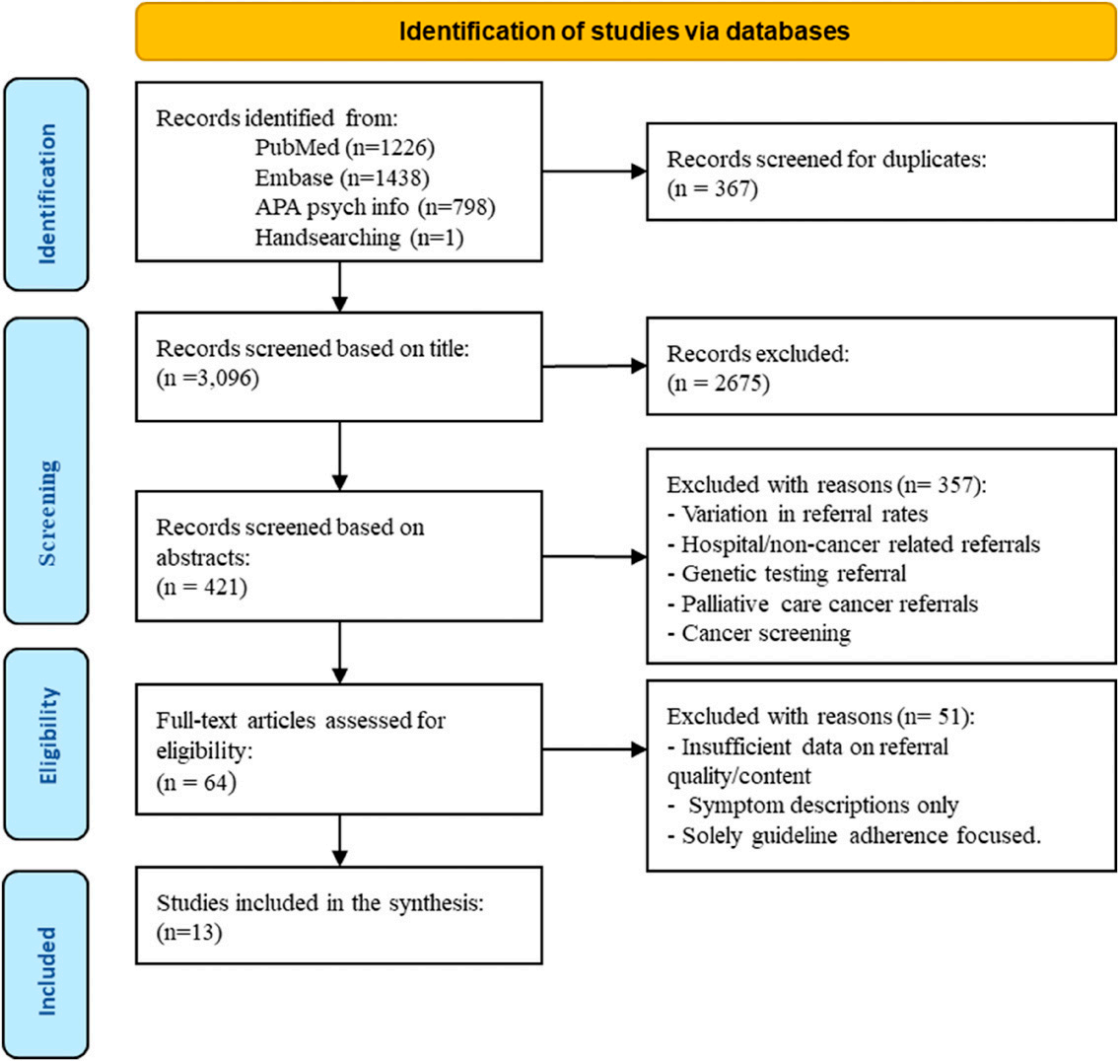
PRISMA Diagram Outlining Study Selection From the Data Sources Included in Literature Search

The database search returned 3463 results. After the elimination of duplicates, 3096 titles were screened. A total of 421 abstracts were reviewed, and only 64 articles were selected for full-text review. Upon full-text review, thirteen met the study eligibility criteria. Figure 1 below outlines the screening process and reasons for exclusion.

The summaries of the thirteen articles included in the review are shown in Online Appendix A. Eleven studies were conducted in the United Kingdom (UK), one in Sweden, and one in Australia. Qualitative research methods were employed in eight studies, using interviews or focus group discussions with primary care clinicians, patients, or both to assess referral form quality, referral reason and content, as well as the referral process. Three studies employed retrospective audits of referral forms via urgent and routine referral pathways, and one used a mixed-method approach. Sample sizes of these studies ranged from 12 to 432 for different types of cancer referrals, including testicular, oral and maxillofacial, oropharyngeal, and head, neck, and breast cancer.

### Critical Appraisal of Selected Studies

We used the Mixed Methods Appraisal Tool (MMAT) for appraising systematic mixed studies reviews, including qualitative, quantitative, and mixed methods designs.^16^ The quality of each paper was assessed using the yes-or-no checklist, and the percentages of their results were calculated as shown in Online Appendix A. Results from the quality assessment show that twelve papers are of high study quality, while the remaining paper has an acceptable study quality. All studies were included in our review.

## Narrative Findings

### Detailed description of patient’s symptoms

Several studies highlighted the importance of detailed symptom description in referral forms to communicate the nature of the patient’s current presenting problem.^17,18^ Kumaraswamy et al. (2009) reported that detailed symptom characteristics such as the nature, duration and progression of symptoms, as well as descriptive characteristics were often omitted. For example, the size of lumps and location of swellings in the scrotum were frequently missing on suspected testicular cancer referrals forms.^19^ Similarly, Patel et al. (2009) audited twenty-eight patients referred by General Dental Practitioners (GDPs) for suspected oral, head, and neck cancer, finding that less than half contained detailed symptom information such as pain on swallowing, deafness, sore throat, hoarseness, and precise location of suspected cancer area.^11^ White et al. (2004) conducted a content analysis of GDPs’ referral forms relating to suspected oral malignancy, which were categorised into two groups by differences in quality. Group A forms included detailed lesion descriptions, risk factors, and management history, while Group B forms only contained basic lesion descriptions.^18^

Two retrospective studies found that the nature of the referral pathway affected symptom description detail. These studies audited referral forms related to the colorectal cancer pathway and compared urgent referral forms to routine referral forms.^20,21^ They both found that urgent forms included a greater volume of detailed symptom information compared to routine referral forms.^20,21^ Jiwa et al. (2007) reported that fast track referrals from primary care clinicians to colorectal surgeons contained more symptom description, signs and associated risk compared to non-fast track referrals.^20^ Furthermore, Dua et al. (2009) found that information regarding changes in bowel habits and iron deficiency anaemia were significantly more likely to have been included in urgent referrals than in routine ones.^21^ In contrast, the mixed methods study by White et al. (2004) reported that 30% of GDPs believed that it was unnecessary to include any description of oral lesions in referral forms, and 40% considered detailed lesion descriptions non-essential.^18^

### Inclusion of Completed Blood Tests and Physical Examinations

The importance of including documentation of clinical examinations in referrals for suspected testicular, colorectal, and ovarian cancer was reported in two retrospective audit studies^19,21^ and one qualitative study.^22^ These studies reported that information about clinical examinations was often missing; for instance, more than half of colorectal referrals omitted clinical examinations completed in primary care, such as digital rectal and abdominal exams.^21^ Among these patients, where evidence of examination was lacking in the referral form, more than one-third (34.1%) had palpable rectal cancer detected in the specialist clinic.^21^ Similarly, frequent omission of relevant information from physical examinations such as scrotum examination, and results from transillumination with a pen torch for testicular cancer referrals led to difficulty in differentiating between cancerous and non-cancerous swelling.^19^ In the qualitative study, primary care clinicians agreed that there was a need to include information about test results completed in primary care such as CA-125 for ovarian cancer, especially for fast-track systems.^22^

### Inclusion of GP Motivation and Subjective Judgement

Some studies reported that it was beneficial for primary care clinicians’ referral forms to include non-clinical information, such as subjective judgement.^17,20^ Two studies suggested that including the motivation behind primary care clinicians’ referrals in documentation could help specialists recognize the urgency and nature of the referral.^17,20^ In particular, one study of GP interviews reported the use of intuition or a “leap in imagination” to foresee what information might be useful to the specialist, going beyond just clinical facts.^17^

## How do Attempts to Structure Forms and Criteria Affect Quality of Referral Forms

### The Role of Template or Proforma in Referral Forms

Two studies reported the use of a proforma in cancer referrals; a structured form including tick-boxes to ensure the inclusion of key information. However, both studies reported that these forms often resulted in prioritisation of a small number of symptoms or features, at the cost of omitting more detailed aspects of individual patient cases.^17,21^ Dua et al. (2009) reported that primary care clinicians were more likely to report prioritised clinical features as a way of warranting referral if using the proforma.^21^ As a result, primary care clinicians preferred referral forms to be customized or tailored to suit the specific needs of individual patients.^17^

### Role of Guidelines in High Quality Referral

We found mixed evidence about the impact of guidelines on cancer referral form quality, based on 3 qualitative studies. Jiwa et al. (2002) reported that primary care clinicians considered guidelines to be too lengthy and numerous, while others appreciated that they facilitated symptom management; thereby complicating their effectiveness.^17^ Since this study in 2002, guidelines in England have changed. Suspected cancer referral guidelines were created in 2005 by the National Institute of Health and Care Excellence (NICE) and updated in 2015. Three studies reported that guidelines created referral barriers for primary care clinicians and specialists when patients’ symptoms did not match criteria.^17,23,24^ Redaniel et al. (2015) reported that primary care clinicians faced difficulties writing referrals for patients with non-specific symptoms that did not meet the guidelines, leading to calls for more flexible referral routes.^24^ A similar qualitative study of primary care physicians’ views conducted in Sweden found that they faced difficulty in identifying diagnoses and deciding what to include in suspected cancer pathways referrals due to guideline restrictions.^25^

Two retrospective studies also indicated high levels of guideline non-compliant referrals, suggesting that they might not have had a strong impact on referral form quality. Kumaraswamy et al. (2009) reported that suspected testicular cancer referrals often deviated from guideline criteria, with 48% of referral forms indicating that the patients symptoms were not aligned and 18% directly violating protocol.^19^ They stated that this resulted in excessive referrals and created additional stress for specialists.^19^ Similarly, Jiwa et al. (2007) reported that only half of referral forms to colorectal specialists for patients with iron-deficiency anaemia were guideline-compliant.^20^

## The Impact of GP-Specialist Relationship on Referral Forms

### GP-Consultant Relationship as a Quality Factor

We found some evidence that the relationship between primary and secondary care practitioners affected the content and quality of referral forms, with qualitative studies indicating that primary care clinicians anticipated judgement of referral forms by specialists as a measure of their performance and consultation quality.^17,23-27^ Jiwa et al. (2002) reported that primary care clinicians often incorporated non-clinical patient insights such as concerns, yet worried that such practices might lead to specialists undervaluing their referral forms.^17^ The authors suggested that feedback from specialists could enhance the quality of their referral forms.^17^ Conversely, participants in 3 studies with primary care clinicians felt that their referral quality had been undermined by a lack of referral confirmation routines, communication issues, and feeling unfairly questioned by specialists.^24-26^ They also suggested that clearer directives and greater collaboration between primary and secondary care providers were needed to accelerate investigations, stating that a stronger relationship between primary care clinicians and specialists would have improved the chances of high quality referral outcomes such as patients being seen quickly, leading to increased adherence to guidelines.^23,24,26^

In addition to improving the referral documents, two studies reported the need for personal intervention on the part of the primary care clinician to enhance referral quality. For example, Pascoe et al. reported Australian primary care clinicians’ belief that direct communication with surgeons to arrange consultations using telephones enabled faster responses and enhanced referral quality.^27^ Similarly, Jefferson et al. reported that primary care clinicians reported booking appointments themselves during consultations and completing online referrals, rather than postponing these tasks until later or assigning them to administrative staff.^28^

## Impact of Referral Form on Diagnostic Outcomes in Secondary Care

There was limited evidence on the impact of referral quality on diagnostic outcomes. Those that reported outcomes used mainly anecdotal or qualitative reports rather than measured outcomes. One study reported that the limited use of clinical photographs and absence of detailed lesion descriptions in referral forms led to delays in diagnosing and treating oral malignancy.^18^ Similarly, where information about rectal exams was omitted in the referral, some patients’ referrals were rejected from the urgent suspected cancer referral pathway.^21^ Patients referred for suspected malignant oral, head, and neck cancer experienced delays in securing initial outpatient appointments for up to 8 weeks due to incomplete referral forms.^11^

## Discussion

This scoping review has synthesised evidence from thirteen studies about the optimal content of referral forms for suspected cancer referral, and its impact on diagnostic outcomes. The included studies reported a moderate amount of qualitative and quantitative evidence relating to referral content. Synthesising the evidence and authors’ comments in the included studies, we suggest that optimal referrals would include:

• detailed symptom descriptions such as size, appearance, duration and other qualitative characteristics
• clinical examination and testing results from primary care such as physical examinations
• non-clinical information such as concern or overall motivation for referral.

The reviewed studies included mixed evidence relating to the impact of templates or proformas on quality of referral forms. We found that while proformas may improve consistency with respect to ‘red flag’ symptoms, they could also result in poorer reporting of other symptoms and clinical information. A minority of studies reported qualitative evidence that omission of symptom information or symptom descriptions in referral forms could delay the diagnostic process to the detriment of quality of care.^10,29^ Finally, our review highlighted that communication and feedback between primary and secondary care clinicians can affect diagnostic outcomes, both in terms of the content of documentation and provision of additional verbal information.

### What this study adds to previous literature

Our main finding is that referral forms for a suspected cancer would be optimised by detailed symptom information including the size, appearance and duration, as well as clinical approaches to investigation that have been completed. The evidence we reviewed pointed to the use of detailed symptom description to enable specialists to understand the reasons for referral, make triaging decisions and assess the urgency of further investigations or interventions.

Our review also suggests that optimal referral content varies by cancer site. For instance, in breast cancer referrals, size, mobility, and fixation of palpable lumps are essential, whereas in oral cancers, where lesions are visible, optimal referrals may include detailed lesion descriptors (eg, ulceration, induration) and, where feasible, clinical photographs. Symptom documentation may also benefit from clearer guidance. For example, weight loss can be described by patients qualitatively, such as noticing looser clothing or receiving comments from friends and family. Because weight is not routinely recorded in many primary care settings—and recording is often biased by comorbidity or incentivisation—quantitative evidence of weight loss is frequently unavailable.^30^ Primary care practitioners should be permitted to document weight loss as it is presented to them, if they deem it clinically significant. Where a reduction in weight has been measured, evidence suggests that the rate of weight loss is more predictive of cancer than the absolute amount or percentage of body weight lost.^30^

Current guidance, including NICE guideline for suspected cancer referral (NG12), does not always specify the level of detail required. Our findings suggest that lack of standardisation leads to variability in referral quality, supporting the case for more explicit prompts in referral proformas and structured educational interventions for referring clinicians. The evidence reviewed suggest specific areas where referral templates might be enhanced. For example, digital forms could prompt structured input for physical examinations or test results, including fields for unstructured commentary where clinicians express concern or provide rationale. These findings could usefully inform future revisions to standardised referral forms or associated tools.

Other studies suggest that this information should not be too burdensome, however. In a related Delphi study, both primary care clinicians and specialists reached consensus that information in referral forms for patients being treated for cancer should be limited to presenting symptoms, history of symptoms, physical examinations, and investigations in order to reduce the burden on secondary care.^31^ Other researchers have suggested that training healthcare professionals may improve accurate symptom documentation in referral forms, enhance understanding of patient circumstances, and improve referral quality.^32-34^ A novelty of our findings is the importance of including non-clinical information such as GP motivation or concern, which appears to contradict the consensus reached in the Delphi study.^31^ McConnell et al. (1999) also demonstrated that the quality of doctor-to-doctor communication in oncology referrals is shaped not only by clinical content but also by tone, clarity, and narrative structure.^35^ This aligns with our finding that non-clinical elements—such as GP concern or intuitive judgement—can aid triage and decision-making.

Our review lacked evidence about the order or prioritisation of information. A qualitative study of correspondence between primary and secondary care found that referrals about lung, colorectal, and breast cancer treatment to oncology tended to contain information relevant to the current problem mixed with less relevant details.^36^ This issue was most notable in the past medical history section, where major and minor health problems were often listed together without prioritization and sometimes without chronological order.^36^

We found mixed evidence about the impact of cancer referral guidelines and templates for patients, particularly highlighting the difficulty for primary care clinicians of referring patients whose symptoms do not match the guideline. A substantial literature has also highlighted the challenge of finding appropriate referral pathways for patients with non-specific symptoms.^37^ Non-specific symptom pathways are now implemented in the United Kingdom and elsewhere in Europe; while there is no current NICE guideline for referral, the inclusion of primary care clinician ‘gut feeling’ as a criterion for referral in service guidance is an important move forward for these patients.^13,38,39^ While gut feeling is often linked to non-specific symptom pathways, it also plays an important role in site-specific pathways where symptom presentation is atypical or borderline for referral criteria.

Our review and other studies have found that primary care clinicians sometimes discuss potential referrals with a specialist over the telephone, even though they typically complete a referral form to clarify these reasons for reasons of accountability and audit.^13,40^ However, this may be particularly relevant to healthcare environments without electronic referral systems, noting that some of the evidence we included was nearly 20 years old. In a 2019 Swedish study, however, both primary care clinicians and specialists mentioned that they preferred telephone contact when dealing with oncological diagnoses in referral forms, despite difficulties relating to mutual availability.^36^ Advice and guidance services in the UK NHS have proven effective in enhancing patient care and streamlining surgical referrals by enabling primary care physicians to consult digitally with specialists.^41^ This type of model may be effective to optimise cancer referrals, offering primary care clinicians a consistent platform to seek specialist input prior to making referrals.

## Strengths and Limitations

Our findings suggest some agreement around content of optimal referral forms for suspected cancer. A strength of our research is the broad scope and relevance to countries with similar cancer referral systems to the UK. However, the findings should be interpreted with caution due to the limited number of studies available. Very few studies in the review explicitly linked referral content and quality directly to outcomes. Another limitation relates to the indexing systems employed by databases to categorize publications, which might have obscured some relevant publications. While PubMed and Embase are extensive academic databases, the primary search terms had numerous synonyms and definitions which might have led to missed relevant literature. Additionally, we were unable to archive our reasons for exclusion at abstract and full text stage due to limitations of the referral software. This limits granularity in our reporting.

### Implications for future research

Future research should focus on directly linking the content of cancer referral forms to diagnostic outcomes. This could involve employing methods such as prospective or retrospective cohort studies, and case studies to examine how specific elements of referral quality such as completeness and clarity of information are associated with key diagnostic outcomes, including time to outpatient appointment, timing and appropriateness of diagnostic testing, as well as clinical outcomes such as diagnosis, stage at diagnosis, treatment initiation, and survival rates. Studies could also explore which referral elements are most valued by secondary care clinicians in triage decisions.

The findings from such research could drive improvements to optimise referral forms for suspected cancer investigation.

## Conclusion

The literature included in this scoping review suggests that optimal referral forms for suspected cancer should contain detailed symptom descriptions, clinical examination and testing information, and indications from the primary care clinician about their rationale to optimise diagnostic investigations. Incorporating these details into cancer referral forms ensures that specialists can make informed decisions about diagnostic investigations, triage and prioritise cases effectively, and minimise the risks of misdiagnosis or treatment delays. Future research should focus on linking referral form content to diagnostic outcomes. Additionally, research into guidelines and proformas should seek to resolve contradictory findings about their impact on diagnostic outcomes, as well as how to make provision for patients with non-specific symptoms.

## Supplemental Material

Supplemental Material - Characteristics of Optimal Cancer Referrals Made by Primary Care Clinicians: Scoping Review Supplemental Material for Characteristics of Optimal Cancer Referrals Made by Primary Care Clinicians: Scoping Review by Olufisayo Olakotan, Judith Yargawa, Julie-Ann Moreland, Claire Friedemann Smith, Brian D. Nicholson, Andrew Millar, and Georgia B. Black in Cancer Control

## Data Availability

The raw data that support the findings of this study are available on request from the corresponding author.*
