# Supplementary material for: Characteristics of Optimal Cancer Referrals Made by Primary Care Clinicians: Scoping Review
Source: Cancer Control. 2025 Jul 28;32:10732748251359405. doi: 10.1177/10732748251359405 (PMC12304610; doi:10.1177/10732748251359405)
Supplement: Supplemental Material - Characteristics of Optimal Cancer Referrals Made by Primary Care Clinicians: Scoping Review [file sj-pdf-1-ccx-10.1177_10732748251359405.pdf]

Appendix A. Characteristics of selected study

**Study Identification**			**Study characteristics**			**Referral characteristics**			**Outcome measures**	**Method used to capture data**	**Quality assessment**	**Findings**
Authors/year		Country	Study design	Sample size	Cancer type	Element of referral form being studied	Referral form aspect linked to quality	How quality of the referral form content were assessed	Measures in relation to the quality of the referral	Electronic or paper form	MMAT Scores in %	Summary of findings
1	White et al, 2004	United Kingdom (UK)	Mixed method (Qualitative/ Content analysis)	65	Oral	Patient demographic (e.g. patient's date of birth, Patient's phone number, address) Referring physician information (e.g. referring GDPs name, address, and telephone number)	Legibility/clarity (Ascertain if referral letters were eligible)	Criteria development and application	Quality of referral and waiting times	Paper based form	75%	Highlighted deficiencies in GDPs' referral letters for potential oral malignancy, especially in lesion descriptions. A lack of detailed lesion description might affect specialists' ability to prioritize cases, leading to potential delays in diagnoses and treatment for serious conditions like oral malignancy.
2	Farquhar Mc et.al 2005	UK	Qualitative study (Interviews)	12	Ovarian	Not mentioned	Relevant test/Investigation results (e.g. CA 125 blood test, ultrasound)	Clear referral criteria	Improve content and format of communications	Telephone	100%	GPs expressed the need for clearer information on tests like CA-125, ultrasound, and the use of the fast-track referral system; underscores the importance of prompt communication and clarity in test usage and referral processes.
3	Michael B et al 2020	Sweden	Qualitative study (Semi-structured)	27	Ovarian	Not indicated	Not indicated	Not indicated	Measure the level of satisfaction among PCPs regarding the SCP pathway and guidelines)	Not indicated	100%	Highlighted challenges in interpreting what investigations should be included in referrals and varying knowledge of standardized care pathway SCPs in primary care units. The study calls attention to the need for well-formulated criteria and clear directives in SCP processes.
4	Sascha et al 2009	UK	Retrospective audit of referral letter	150	Colorectal	1) Patient demographics (e.g.70 were female, 80 were male with a median age of 69 years (range 24–88) 2) Clinical presentation (e.g. change in bowel habit, PR bleeding, Iron deficiency anaemia)	Relevant test/investigation results (e.g. clinical examination such as abdominal or rectal examination)	1) Peer review 2) Clear referral criteria/ checklist	Impact of the two referral modes TWR and non-TWR on time to treatment	Electronic (EHR)	100%	Many referral documents lacked complete clinical information, and there was no significant difference in referral routes based on symptoms like rectal bleeding or altered bowel habits. Emphasizes the need for more effective use of referral criteria and thorough clinical examinations.
5	Green et-al 2015	UK	Qualitative study (semi-structured)	55	Not indicated	Not indicated	Not indicated	1) Referral criteria/guidelines	Improve cancer diagnosis	Not indicated	100%	Focuses on the limitations of 2-week-wait (2WW) urgent referral routes when symptoms do not meet guideline criteria. It highlighted GPs' strategies to overcome these barriers and the evolving nature of the primary/secondary care relationship in cancer care.
6	Laura Jefferson	UK	Qualitative (Individual	20	Head & neck,	Not indicated	Not indicated	Not indicated	Understand reason for non-	Not indicated	100%	It highlighted the challenges GPs face in managing appointments for suspected cancer referrals and the

	et-al 2019		interviews)		breast				attendance for appointments			complexities of doctor-patient communication in this context. GPs, however, made real-time online referrals. Despite a consensus-developed patient leaflet for the 2WW pathway, only a third of GPs used it.
7	Maria Theresa et-al 2015	UK	Qualitative (Semi-structured)		Colorectal	Clinical presentation (e.g. vague symptoms, such as unexplained weight loss, right lower abdominal mass rectal bleeding, or weight gain)	Not indicated	Referral criteria	Recommendations for Pathway Improvements	Not indicated	100%	This research focused on the difficulties in applying the TWW referral criteria for colorectal cancer, especially with non-specific symptoms and comorbidities. It pointed out the variations in referral practices and the tension between adhering to guidelines and using professional judgment.
8	Mark Harris et al 2012	Australia	Qualitative (focus groups)	55	Colorectal	Not indicated	Not indicated	Not indicated	Improving communication between GP and surgeon facilitate referral pathway	Not indicated	100%	GP prefer direct phone contact for CRC patient referrals due to faster responses, but time constraints make it challenging. Delegating the task was common, yet personal GP involvement often led to higher quality referrals. The stronger the relationship between GP and surgeon, the quicker and better the patient outcome.
9	Mital patel et-al, 2011	UK	(Retrospective audit of form)	58	Oral and Maxillofacial	1) Patient's Demographic Details (e.g. DOB, age gender, NHS number, Language spoken)<br>2) Referring physician's information (e.g. referrers name, address, tel no, fax no)<br>3) Clinical presentation<br>4) Level of urgency	Not stated	Content analysis	The time taken from the point of referral to an outpatient appointment being made	Paper based form	100%	The study found that out of twenty eight patients suspected of having malignant oral, head, and neck cancer, only twelve referrals contained adequate details for quick consultant triage. Clear referrals include clinical symptoms, suspected cancer location, and clinical findings. Incomplete referrals caused appointment delays for sixteen patients, emphasizing clear referral's importance.
10	Moyez jiwa et-al 2002	UK	Qualitative study (semi-structured)	12	Colorectal	Clinical presentation (e.g. bowel cancer symptoms often resemble benign diseases)	Comprehensive clinical information (e.g. medical history," and other relevant details such as "medications they're on, allergies and any)	Clinician feedback	Improve the quality of GP referrals for colorectal cancer	Paper based forms	100%	Referral letters should include foundational details like medical history and current issues for specialists' understanding, including medications, allergies, and social factors. GP's subjective judgment in referrals involves balancing essential details without overwhelming, anticipating the specialist's needs, described as a "leap in imagination.
11	Moyez Jiwa et-al 2007	UK	Retrospective audit of letters	432	Colorectal	Clinical presentation (e.g. duration of symptoms, rectal mass or results of rectal examination, abdominal mass)	1) Clear statement of referral reason<br>2) Relevant test/Investigation results (e.g. full blood counts, abdominal examination)	Referral guidelines	Cancer diagnosis in urgent referrals	Paper based forms	100%	The study analysed GP referral letters to colorectal surgeons. Despite guidelines, only half with iron-deficiency anaemia got urgent colonoscopy referrals. Fast-track referrals were more detailed, while non-urgent ones often lacked clinical details. Despite some referrals hinting at urgency, none of the routinely referred patients were diagnosed with cancer.
12	P. R. Brocklehurst et-al 2009	UK	Qualitative study (Interview study)	18	Oral Oropharyngeal Cancer	Clinical Presentation (e.g. Ulceration, Indurated borders, Fixed lesion, Red or speckled appearance)	Comprehensive clinical information (e.g. patients risk factors)	Emergent theme and codified information derived from the interview	Decision to refer	Paper based forms	100%	Real concern about a lesion for many dentists would prompt immediate action to contact secondary care directly. A number of factors that can have an impact on the detail of the referral process were also cited by the participants, including the PCDs' confidence and their relationship with the referring hospital
13	Prakash kumaraswamy et-al 2009	UK	Retrospective study	241	Testicular cancer	Patient's demographic details (e.g. age range was 12–87 years of age (average, 42 years)	Relevant test/ investigation results (e.g. Ultrasound)	Audit and feedback	Compare waiting times from referral to treatment after introduction of	Paper based forms	100%	The study carefully examined the notes and referral letter for evidence of a previous vasectomy. It is clear that the referring GP rarely mentioned such a history when this was identified by the urologist and it was obvious that hardly any GPs appreciated that vasectomy can cause

						Clinical presentation (e.g. scrotal swelling, intermediate swelling and solid swelling)			two weeks wait			swelling of the epididymis or other masses such as a palpable vasectomy site or sperm granuloma

Appendix B. Search terms used in bibliographic database

Search Results	Search strings
PubMed	("General Practice"[MeSH] OR "Primary Nursing"[MeSH] OR "Preventive Health Services"[MeSH]) AND ("Referral and Consultation/standards"[MeSH] OR "Appropriateness"[MeSH] OR "Time Factors"[MeSH] OR referral qualit* OR referral appropriat*) AND ("Neoplasms"[MeSH] OR tumor* OR "Neoplasms"[MeSH] OR cancer* OR malignan* OR "Carcinoma"[MeSH] OR "Sarcoma"[MeSH] OR "Lymphoma"[MeSH] OR "Melanoma"[MeSH]) AND ("Referral and Consultation" OR inappropriat* OR Referral quality* OR Referral letter* OR late OR unsuitab* OR unnecessar*))
Embase	('quality improvement'/exp OR 'quality assurance, health care'/exp OR 'quality control'/exp OR 'health care quality':ab,ti OR 'healthcare improvement':ab,ti) AND [english]/lim AND ('neoplasm'/exp OR 'carcinoma':ab,ti OR 'sarcoma':ab,ti OR 'leukemia':ab,ti OR 'lymphoma':ab,ti) AND [english]/lim AND ('primary health care'/exp OR 'family practice'/exp OR 'general practice'/exp OR 'primary care nursing'/exp OR 'primary care':ab,ti OR 'family medicine':ab,ti OR 'general practice':ab,ti OR 'GP':ab,ti) AND [english]/lim AND ('referral and consultation'/exp OR 'referral quality':ab,ti OR 'referral appropriateness':ab,ti OR 'referral letter':ab,ti OR 'referral form':ab,ti) AND [english]/lim
APA PyscInfo	("general practitioners" OR "primary care physicians" OR "family doctors" OR "family physicians" OR "primary care providers" OR "primary healthcare professionals" OR "general practice physicians" OR "family medicine practitioners" OR "GP" OR "primary care clinicians") AND ("quality" OR "appropriateness" OR "timeliness" OR "suitability" OR "necessity" OR "referral quality" OR "referral appropriateness" OR "referral timeliness" OR "referral suitability" OR "inappropriate" OR "late" OR "unsuitable" OR "unnecessary" OR "delay" OR "referral" OR "referral rate" OR "variation") AND ("cancer" OR "neoplasm" OR "tumor" OR "malignancy" OR "oncology" OR "carcinoma" OR "sarcoma" OR "leukemia" OR "lymphoma" OR "melanoma")

	Author, year	Brief description of methodology	Study aim	Thematic findings	Verbatim Quotes in Italics/Additional notes & Summary
1	White et-al 2004	1) Firstly, referral letters from Birmingham General dentist practitioners (GDPs) to the Oral Medicine service in the quarter. As the main emphasis of the study was to ascertain practitioners' views on the referral process, a 3-month period was deemed sufficient to identify a range of referral letter quality and therefore an appropriate sample of GDPs. Criteria for categorising the letters were developed based on both previously published work and the particular needs of the service<br>2) During the second phase, qualitative research methodology was used in order to elicit in-depth information from a sample of the GDPs whose letters had been reviewed in the first stage. A purposive sample of practitioners was chosen to represent a range of views from those who did and did not meet the referral criteria, rather than a statistically representative sample and overall, twenty practitioners were selected (ten from group A and ten from group B).	The initial phase aimed to assess the quality of referrals from GDPs for patients with a possible oral malignancy. Subsequently, qualitative methodology was used to ascertain GDPs views of the referral process for patients with oral mucosal lesions and factors impacting on this. The purpose of this type of research is to give a deeper explanation and understanding of a problem rather than to quantify it.	**Referral letters:** A total of 64 relevant referral letters received by the Oral Medicine service between October and December 2000 were identified. Applying the referral letter criteria resulted in approximately equal numbers being allocated to group A (30) and group B (34). However, all referral letters from both groups had scored highly in stage one, with almost all including referring practitioner and patient details. Two thirds of letters were either typed or word processed and of those hand-written, only two were difficult to read. The main difference between the two groups was in their description of the lesion. Whereas all practitioners in group A had included a detailed description of the lesion, management to date and in some cases, risk factors, those practitioners in group B had only provided a very basic description.<br>**Referral information:** The qualitative data provided points to notable deficiencies in the quality of referrals from GDPs for patients with potential oral malignancy. The deficiency mainly lies in the lack of detailed descriptions and documentation of the lesions, and the limited use of clinical photography to support the referral. On the other hand, a considerable number of practitioners (30%) did not believe that including any description of the lesion was essential, and 40% did not consider a detailed description essential. This lack of lesion description might affect the ability of specialists to prioritize cases effectively, potentially delaying diagnoses and intervention for serious conditions like oral malignancy.	1) The qualitative data provided points to notable deficiencies in the quality of referrals from GDPs for patients with potential oral malignancy. The deficiency mainly lies in the lack of detailed descriptions and documentation of the lesions, and the limited use of clinical photography to support the referral. On the other hand, a considerable number of practitioners (30%) did not believe that including any description of the lesion was essential, and 40% did not consider a detailed description essential. This lack of lesion description might affect the ability of specialists to prioritize cases effectively, potentially delaying diagnoses and intervention for serious conditions like oral malignancy.<br>2) The quantitative data indicates that the referral letters received by the Oral Medicine service were generally of high quality in terms of inclusion of essential basic details like referring practitioner and patient information. There is, however, a discernible difference in quality between the two groups (A and B) based on the description of the lesion provided. Detailed Description of Lesions in Group A: Referral letters were notably superior in terms of providing detailed descriptions of lesions, management to date, and, in some cases, risk factors. This detailed information is essential for appropriate triage, efficient management, and tailored patient care. Lack of Detailed Description in Group B: Conversely, the referral letters in Group B were suboptimal in the quality of clinical information provided, offering only very basic descriptions of the lesions. This lack of detail could hinder effective and timely management of the patients, leading to potential delays in diagnosis and treatment.
2	Mital Patel, 2011	Audit of database, names of all patients who were newly diagnosed with oral and oropharyngeal cancer during a two-year period (2007–2009) were obtained at the Oral and Maxillofacial Department at Barnet and Chase Farm NHS Trust, to examine the quality of referral letters sent for potentially malignant oral, head and neck lesions.	The aim of this retrospective audit was to assess the quality of referrals sent to our department for potentially malignant oral, head and neck lesions and to analyse how the quality of the referral letter affected the time taken from the point of referral to an outpatient appointment being made.	1) A total of 75 patients were diagnosed with oral, head and neck carcinoma during the two-year period. Of these patients, 17 patients were diagnosed during their regular follow-up visit and were therefore excluded from the audit. A total of 58 referral letters were analysed. Of these, 30 patients were referred using the North London Cancer Network (NLCN) referral proforma. Most of these referrals were from GMPs. All the NLCN referrals were treated as urgent by the administrative staff and outpatient appointments were made for the patients without the need for prioritization of the referral by the consultant. The average time for outpatient appointment for patients referred through the NLCN pathway was 12 days, with a range of 10–16 days. The diagnosis, confirmed by a biopsy, was made within 22 days, with a range of 17–30 days.<br><br>2) Twenty-eight patients were referred using a typed or handwritten letter. These letters required prioritization by the consultant prior to an outpatient appointment being made. Amongst these letters, 12 had most of the details required to help the consultant to triage the letter as urgent and thus attempt to give an outpatient appointment within two weeks of the referral. In patients whose letter did not have the urgency highlighted or a suggestion of cancer, the letters were triaged as routine, and an outpatient appointment was given within 6–8 weeks. The average time taken for an outpatient appointment for patients referred using a typed or handwritten referral letter with the appropriate information was 21 days, with a range of 14–30 days.	1) The study reveals that among twenty-eight patients suspected of having malignant oral, head, and neck cancer, who were referred using typed or handwritten letters necessitating consultant prioritization before scheduling an outpatient appointment, only twelve contained sufficient details. These comprehensive details enabled the consultant to quickly triage the letters as urgent and endeavour to arrange outpatient appointments within two weeks of receiving the referral. To clarify, a well-detailed referral in this context includes both crucial clinical and administrative data, properly marked as urgent. This entails clear referral information highlighting symptoms such as pain on swallowing, deafness, sore throat, and hoarseness.<br><br>2) It also specifies the suspected area of cancer, for example, the oral cavity, thyroid, or pharynx, and includes findings from clinical examinations like the presence of a lump in the neck or thyroid. Regrettably, sixteen patients experienced delays in securing their first outpatient appointment due to incomplete or insufficient information in their referral letters, indicative of lower-quality referrals. This study underscores the critical importance of comprehensive and clear referral information in facilitating prompt consultant reviews and timely outpatient appointments for patients with suspected malignant conditions.

				Confirmation of diagnosis for these patients was done within five weeks, with a range of 24–40 days. A total of 16 patients, whose letter did not have the necessary information, had a delay in the first outpatient appointment of up to eight weeks, with a range of 6–10 weeks. Once patients were seen in the clinic and suspected of having cancer there was very little variation, amongst the groups, in the time taken to diagnose and treat the patient.	
3	R.Sascha et al 2009	This study is based on prospective database of colorectal cancer patients, referred by their general practitioner to the North Middlesex University Hospital, via the two week rule TWR and non-TWR pathways, after the introduction of the TWR proforma. Patients were divided into two groups: those referred under the 'two-week rule' criteria (TWR group) and those referred electively to the specialist clinic outside the two-week rule pathway (non-TWR group). Clinical indications for referral, as listed in the TWR proforma and/or primary care referral letter, were compared between the two groups of patients.	This study was conducted to investigate the difference in presentation between patients referred via the TWR pathway compared to those referred via an elective (non-TWR) route and to examine the impact of these referral routes on the time to treatment and clinical outcome.	**Completeness of information provided:** Many referral documents (both TWR and non-TWR) contained incomplete clinical information. In this section of the study the presenting clinical feature(s), mentioned in the referral documents, were recorded. All positive and negative responses to each clinical feature were noted. Where the clinical feature was not mentioned in the referral document, it was assumed that the patient had not been questioned about it. A total of 94 referral documents (62.6%) indicated that the patient had been asked about change of bowel habit, 105 patients (70%) had been questioned about rectal bleeding and 76 patients (50.6%) had been tested for iron deficiency anaemia. Information regarding change in bowel habit (p ¼ 0.0007) and iron deficiency anaemia (p¼ 0.0033) was significantly more likely to have been included in the referral document in the TWR group compared to the non-TWR group. There was no significant difference between the groups on information provided regarding rectal bleeding.<br>**Clinical referral criteria:** Analysis of the referral documents, based on positive responses to clinical referral criteria alone, revealed that all of the 75 patients referred under the TWR, had met at least one of the referral criteria listed in the NHS TWR guidelines. However, of those referred as non-TWR, 69 (92%) would also have met at least one of the criteria for referral under the TWR protocol. The two groups are compared with respect to three cardinal TWR referral criteria. The most common presenting symptom was rectal bleeding (n¼ 68 patients; 45.3%) with patients evenly divided between the two referral groups (32 in TWR group, 36 in non-TWR group, n.s). Accordingly, there was no significant difference between the two groups when using altered bowel habit as a discriminator. Therefore, patients presenting to primary care with rectal bleeding or change in bowel habit were equally likely to have been referred via the TWR or non-TWR routes. The only clinical finding that was significantly different between the two groups was the presence of iron deficiency anaemia (p ¼ 0.0347). Therefore, patients were more likely to be referred using the TWR proforma in the presence of iron deficiency anaemia.<br>**Physical examination:** Physical examination In 68 of 150 patients (45.3%), there was no evidence of a clinical examination (abdominal and/or rectal) having been performed at all. Evidence of a rectal examination, in the referral document, was found in 43.3% (n ¼65/150, 32 non-TWR, 33 TWR) in similar number in the two groups (Table 4). In the non-TWR group, of 6 patients referred with ''piles'' 3 were found to have rectal tumours on examination by the specialist. Two non-TWR patients had pain on rectal examination and the procedure had been abandoned by the referrer. Both of these patients were subsequently found to have rectal tumours. All 8 patients (all in the TWR group) referred with a palpable rectal mass were confirmed to have rectal tumours by the specialist.	1) The Department of Health TWR pathway was designed to expedite referral of patients meeting specific criteria to specialist units in order to diagnose cancers at an earlier disease stage. This study has shown that when patients present in primary care with clinical features suspicious of colorectal cancer, i.e., prolonged rectal bleeding or change in bowel habit, they are often not referred via the TWR pathway to a specialist unit. These patients are equally likely to be referred as routine cases. However, the presence of iron deficiency anaemia prompted referral via the TWR pathway in a significantly higher number of cases. Therefore, an abnormal test result enables case selection more readily than an assessment based on clinical presentation. All the patients in the TWR group presented with at least one cardinal symptom that met criteria under the TWR guideline.<br>2) Clinically relevant information regarding change in bowel habit and iron deficiency anaemia, but not rectal bleeding, was more likely to be included in the referral if the TWR proforma was used. However, in addition to the particular clinical feature that warranted referral as a TWR, many referral forms were lacking in clinical information. This is despite the provision of a tick-box type of proforma designed for ease and speed of use. Therefore, a more efficient use of the TWR proforma is recommended, based on the results of this study. This is expected to improve selection of patients for referral via the TWR pathway.<br>3) In almost half of all cases (45.3%), there was no documented evidence of a clinical examination (abdominal or rectal examination) by the referring clinician. In more than half of all referrals (56.7%) there was no evidence that a digital rectal examination had been carried out. More than one-third of these patients (34.1%), where evidence of PR examination was lacking, had a palpable rectal cancer detected in the specialist clinic. The omission of a digital rectal examination and/or the inability to detect palpable rectal cancers in primary care led to a failure to refer many patients via the TWR pathway. These findings emphasize the need for education at the primary care level to highlight the importance of rectal examination in patients who present with colorectal symptoms.

4	Moyez Jiwa,2002	A convenience sample of twelve GPs was interviewed in Nottinghamshire and inner city Sheffield practices	The aims of this research are to explore the factors that may influence GPs in writing the referral letter when consulting patients presenting with lower bowel symptoms.	**1. Referral letters**<br>**Content of the referral:** The mention of routine information such as a summary of the presenting problem and previous medical history indicates the basic expectations for referral letters. This foundational information is critical for specialists to understand the patient's current situation and background<br>**Subjectivity in letter writing:** The referral process isn't just about conveying clinical facts. The GP's subjective judgment plays a role, balancing between providing sufficient information without overwhelming the recipient. The mention of a "leap in imagination" suggests that GPs sometimes need to interpret and anticipate what the receiving specialist might find most pertinent<br>**Referral Letters as Indicators of GP Performance:** While most GPs seem to believe that the referral letter isn't an accurate representation of their overall performance or the quality of consultation, there are exceptions.<br>**Quality of Care and Referral:** The essence of a referral goes beyond the letter itself. The decision-making process about who to refer, for what condition, and to which specialty is foundational. A well-written letter is just the culmination of this process. Additionally, motivations behind referrals vary; some might be out of convenience, while others are a result of diligent patient care.<br>**Feedback and Consultant's Perspective:** The perceived value of referral letters varies. Some GPs feel that specialists might not give much weight to their letters. However, the desire for feedback suggests that GPs value external opinions on their referrals, indicating a willingness to improve and adapt.<br>**View on Tick Box Letters:** While structured or standardized ("tick box") letters might seem efficient, they may not always capture the nuances of individual patient cases. GPs seem to value the ability to personalize and tailor their referral letters to the specific needs and circumstances of each patient.<br>2. **Colorectal cancer**<br>**Recognition of Urgency/ Accurate Symptom Interpretation:** GPs understand the importance of urgent referrals when cancer is suspected. The promptness of action when faced with potential cancer symptoms is crucial. The challenge of distinguishing between symptoms of colorectal cancer and benign conditions underlines the importance of accurate interpretation. High-quality referral criteria would provide clarity in differentiating between these symptoms.<br>**Guidelines for referrals**<br>Opinions on the role and utilization of guidelines varied among the GPs interviewed. A common sentiment was that there were too many guidelines and they were overly lengthy, making them cumbersome in practice. Dr. Sh., a female GP, expressed the challenge, saying, "They're hard to store and even harder to locate when needed." However, some GPs viewed guidelines positively. Dr. D. mentioned, "Using a protocol makes management easier when everyone follows the same guidelines." While there was an acknowledgment of the existence of guidelines for almost everything, as noted by Dr. P., a female GP, there were also concerns about their limitations. Dr. A., a male GP, shared an instance of a patient in hospice with cancer who didn't fit the standard criteria but was indeed diagnosed with a malignancy.	.Quote: *"For example: 'medications they're on, allergies and any other relevant factors such as social factors* (Dr R., male)'."<br>. Quote:: *'Enough information but not too much' (Dr S., female)." "A process which was also described as 'a sort of leap in imagination'* (Dr Bh., female)."<br><br>Quote*: Dr. G's perspective implies that a poorly written referral could reflect a GP's overall competence or attention to detail.*

5	Moyez Jiwa 2007	Firstly, a prospective analysis of 712 GP referral letters from hospital in Doncaster and Sheffield area. Secondly a self-administered postal survey of GPs. The study reported data for consecutive referrals to colorectal surgeons in South Yorkshire, UK. Data were collected from hospital medical records and referral letters.	To review the assessment of patients as documented in general practitioners' (GPs') referral letters for urgent and routine referrals to colorectal surgeons	A total of 716 consecutive referrals were identified. Of these, 432 referrals were available with data for at least two measures of interest, namely route of referral, referral letter and/or diagnosis. The hospitals in these localities did not require referrals to be sent in any specific format and a variety of referral document types were noted ranging from typed letters and hand-written notes to referral proformas. Eight colorectal cancers were diagnosed. In this period one might have anticipated 45 cancers over six months in this population, i.e. one per GP per year. Of the cases where both diagnosis and route of referral were known, 168/432 (39%) referrals were made on the fast track (i.e. possible cancer), of these 2.6% had cancer. Less than 1% of the rest were diagnosed as cancer. Therefore, cancer was a relatively rare diagnosis even among the cases that were identified as possible cancer or as being of practitioner concern. However, only 50% of cases with iron-deficiency anaemia (14/28) were referred urgently despite the guideline recommendations, and the reason why one in five patients was referred on the fast track could not be surmised from the details recorded in the letters. Letters about patients referred on the fast-track system listed more signs, symptoms and risk factors than letters describing patients referred routinely. However, letters about patients on the fast track where this was not 'merited' contained fewer relevant clinical details than letters about patients on the routine pathway where this was not 'merited'. None of the patients sent routinely, when the guidelines suggested an urgent referral was necessary, had cancer.	1) The retrospective study examined the GP's documentation in referral letters for both urgent and routine referrals to colorectal surgeons. Results showed a clear gap in adherence: only half of the patients with iron-deficiency anaemia were urgently referred for colonoscopy, despite guideline recommendations. Furthermore, the rationale for fast-tracking one in five patients was not clear from the letters.<br>2) In comparing the referral letters, those written for patients on the fast-track system tended to be more comprehensive, outlining a wider range of signs, symptoms, and associated risks than standard referral letters. Conversely, when fast-tracking was deemed unnecessary, these letters often contained fewer pertinent clinical details than some of their standard referral counterparts. Notably, even though some guidelines indicated urgency, none of the routinely referred patients received a cancer diagnosis
6	Farquhar Mc et.al 2005	This study describes general practitioners (GPs') views of the communication issues across the primary/secondary interface in relation to ovarian cancer patients using qualitative interviews with purposively sampled GPs and an audit of hospital medical records of 30 deceased ovarian cancer patients	The study sought to recruit GPs with experience of caring for a patient with ovarian cancer through to the end of life, where the death had occurred within the previous 6 months.	**Diagnostic and pre-diagnostic stage:** At the pre-diagnostic stage, the GPs reported that they needed results of tests communicated to them promptly to aid appropriate referral. Once secondary care reached a diagnosis, the GPs needed to be informed in order to provide support and consider future care. However, a key feature of discussions surrounding communication at this stage of the journey included doubts about the appropriate use of tests and referrals. Several wanted clearer information on the use of the CA-125 blood test, ultrasound and the appropriate use of the fast-track referral system. The diagnostic uncertainty of ovarian cancer, combined with the pressure created by the fast-track referral system and the need for such referrals to be appropriate, was a source of concern for the GPs interviewed.	**Role of CA-125:** '[. . .] *we have now got access to CA-125, the blood test. Having said that, I still think we are all a bit resistant to using it because I don't think the publicity . . . you know, I don't know how much it costs, I have no idea of about anything like that or what the criteria are [. . .], what false positives you will get . . .'* (GP01: 104–115)<br>**Role of ultrasound:** *'[. . .] the problem is if you think you've got somebody with ovarian cancer then yes, you can go with the two-week wait. [. . .] But many times we will examine a woman or do something, and you think, "well that's a bit bulky [. . .]. Is it a fibroid uterus? Is it an ovarian cyst? Is it this?" and you think "either I've got to do a two-week wait", you know, "I think its cancer" and it turns out to be a fibroid uterus or a normal size uterus which we cannot feel properly, and you feel a complete idiot. Or you have to do a routine scan which takes 3 months at least and all you want is a simple answer*.' (GP01: 43–84)<br>**Appropriate fast-track referral:** *'[. . .] there is a certain reluctance to use that [service] unless there is a fairly high degree of suspicion because you get [. . .] some fairly stinging letters coming back to people saying "this was not an appropriate*<br>**fast track referral".** *[. . .] the worry is that if there is a problem then fine, educate and get that problem put right. We are all open to that. What you do not want to do is fire off a letter like that and discourage people [. . .] because you will actually then [. . .] lose the appropriate referrals*.' (GP10: 175–200; 391–398)
7	Mark F Harris et-al 2012	An open-ended focus group guide was developed. Discussions were recorded using a digital recorder and field notes were made during and after the focus	This study aimed to explore GPs' views on the pattern and factors influencing the referral of CRC patients following initial	**GP role in the referral**<br>**The referral process**<br>General practitioners described a range of methods that they used to	*Some GPs delegated this task to others in the practice: ' I get the nurses … it's a waste of my time farting around on the end of the phone.'* [QLD rural GP, female]

		groups	diagnosis with CRC and some of the issues involved in their continuing care after referral	contact surgeons to arrange consultations for patients diagnosed with CRC. Direct telephone contact was preferred because it was more likely to result in a rapid response. However, telephone referrals were difficult due to the number of referrals and limited time in an already crowded general practice work day.<br>The waiting time between referral and securing a booked appointment with the surgeon was critically important to the GPs because of patient anxiety about their diagnosis, especially where there had already been delay in the diagnostic process. This was variable and the source of considerable frustration and uncertainty to GPs:<br>When GPs took responsibility themselves for initiating communication (rather than delegating this to a staff member), they felt that a 'quality' referral was more likely to occur<br>The nature of the relationship was important:<br>The stronger the relationship between GP and surgeon, the more likely was it that the patient would be seen quickly and would have a good outcome:	*'Then we can't determine how long you're going to be waiting ... before this is done.'* [SA urban GP, male]<br>*'You've got to pick up the phone and ring the surgeon "mate, what's happening there"?'* [NSW urban GP, male]<br><br>*'Strike up a referral relationship with one of them so we ... feel that we can get a good deal from them so occasionally when you do ring them up and say "look this guy really does need to be seen quickly like within a week or two" ... they say "fine send them down".'* [QLD rural GP, male]
8	Laura Jefferson, 2019	Qualitative study in GP practices in one Northern English city. In-depth, individual interviews were undertaken face-to-face or by telephone between December 2016 and May 2018, followed by thematic framework analysis.	To examine how interpersonal, communication, social, and organisational factors influence a patient's non-attendance.	**Referral Process**<br>GPs described struggling to undertake appointments involving suspected cancer referrals in 10-minute timeslots. This explains why online referrals were either completed at the end of a clinic or by practice administrators. A few GPs completed the referral online themselves during the consultation and, in some instances, completed a choose-and-book appointment booking with the patient:<br><br>Within the city in which this study took place, an information leaflet had been developed for patients being referred on the 2WW pathway. It had been agreed between the hospital trust, local clinical commissioning group, practices, and a patient representative group, but only 7 out of 21 participating GPs used it. Two patients commented that they would have found a leaflet useful. The online 2WW referral includes a prompt to ensure the GP has given this leaflet, however, since GPs rarely complete the referral process during a consultation, this may not be done. Some GPs deliberately did not give the leaflet as they felt the reference to cancer would worry patients:<br><br>**Doctor patient communication**<br>Many GPs commented on how conversations with patients had become more difficult as National Institute for Health and Care Excellence guidance for certain cancer referrals had lowered referral thresholds. GPs believed this may dilute messages given to patients and held concerns about increased pressure on hospitals:	*'So you can actually do [a choose-and-book appointment] on the system with the patient in the room and they walk out with the date ... I personally do that, but I'm the only one in my practice that does, my colleagues use our secretaries.'* (GP22, M, age 40 years, 12 years' experience)<br><br>*'It's treading that fine line, isn't it, between wanting them to know it's important they get followed up and not wanting to scare.'* (GP36, F, age 43 years, 16 years' experience)<br><br>*'Basically you can't get an ultrasound scan and you can't get gastroscopies. We've had MRIs now stopped for GPs ... because the whole system has been swamped by this 2-week increased guidelines.'* (GP10, M, age 45 years, 16 years' experience)<br><br>*There was a sense that referral thresholds were often lower among more recently qualified GPs, described as a 'tick-box generation'. Some GPs suggested that a growing fear of litigation* (GP05, GP08, GP21) *may create 'soft' referrals, used to reduce uncertainty.*
9	Michael Bells et al 20202	In total, 27 semi-structured interviews (17 individual and 10 group interviews) were conducted within 24 primary care units, including 61 physicians representing the public and private sectors.	The aim of the current study is therefore to examine PCPs views of implementing standardised care pathways (SCPs) in cancer care.	**Challenges related to new referral procedures:** Participants identified challenges and uncertainties related to what a referral concerning the investigation of SCPs should contain in the case of a well-founded suspicion of cancer. One aspect concerned which investigations to conduct. The participants stated that there was a challenge in interpreting what investigations would be done within the respective cancer diagnosis before the patient was referred to specialist care and that there were difficulties in determining which diagnoses should be referred via SCPs. Another challenge concerned the varying level of knowledge regarding SCPs in most primary care units, which was a problem as the individual physicians' previous	**Quote 1:** [*You do not encounter every type of cancer each year, so there's definitely a risk there.*]<br>**Quote 2:** [*I don't know what procedures other clinics have. We don't know this when we send referrals. Do they look at them the same day or maybe it's twice a week they check for incoming referrals? It is a bit of a concern. I can't let it go. I end up following it, thinking 'come on, do something'. But maybe they've planned it and so on, but it is not written down anywhere, so it gets a bit... It would be good to know how quickly they look at them*.<br>Quote 3: [*'' I haven't read through everything at once, but*

				experience with cancer patients was described as crucial in finding and identifying future cancer patients. At the same time, the participants stated that they rarely meet cancer patients and for this reason have difficulty getting used to this way of working.<br>**Communication related issued:** However, there were participants who were more critical about the cooperation between PCPs and specialist care. The challenges of the collaboration were described as a lack of communication, a lack of routines for referral confirmation, and that they felt specialists were questioning them on issues where they felt there should be consensus. This in turn negatively affected the referral procedures. The participants expressed a desire for all professionals to look beyond their own activities and for primary care to have clearer insight into the working methods and referral procedures among their collaborating partners in specialist care. A need for increased consensus on important components to be able to ensure faster investigations, and clearer directives on the referral procedures was requested.<br>**Access to well formulate criteria to make decisions on suspected cancer:** The participants emphasised the importance of having access to the criteria to be able to make decisions about suspected cancer. They emphasised that the already well-known web support VISS [an internet based information tool] was useful for finding information regarding the criteria for malignancy and starting treatment. However, there were some participants who felt that the texts in the web support were too long and difficult to interpret, and they thought that the text on the SCP process could be concretised and made clearer.	*when I read through and looked at the criteria, I found that they are often very simple, well-formulated and not too long [extensive], which makes sense*
10	T Green et-al 2005	Individual face-to-face semi-structured interviews were conducted with 55 GPs from the North and North East of England and Greater London.	To gain an in-depth understanding of cancer diagnosis from the perspective of GPs.	**Referral.** Participants relied on guidelines and the 2-week-wait (2WW) urgent referral routes available for potential cancer symptoms in England (National Institute for Health and Clinical Care Excellence (NICE), 2005). Although GPs valued 2WW, they also highlighted its limitations when symptoms do not meet guideline criteria, and referral criteria then acted as a barrier. Several GPs called for a generic route for suspicious symptoms. GPs had strategies to overcome some of the barriers, although this situation was managed more easily when there were opportunities for dialogue with secondary care colleagues. Participants perceived that the primary/secondary care relationship had changed as cancer care at secondary level became more specialised.	*If somebody comes in with barn door red flag symptoms, you're like yes, this is easy, we've got a lovely [2WW] form, tick all the right boxes and it's quite good because the form kind of makes you, concentrates you on getting the appropriate symptoms, so that's good too. Erm, you put that package together, and off they go and they've got their appointment within two weeks, so for those, the services where we've got that option, and where it's clear cut, it's great.* (GP5/F/2)<br>*Someone comes in coughing up blood, not difficult, er, someone comes in with sort of vague symptoms that turn out to be lymphoma or pancreatic cancer, difficult, you know.* (GP39/M/8)<br>_ *All my patients who are on the two week wait are seen within two weeks, they're seen the next day. I think it's so popular with the health profession, certainly with GPs, I don't know how secondary care feels, because they're the ones under pressure so they're the ones who've got to keep on top of it, so I don't know how they feel about two week wait but certainly from a GP and patient perspective, it's amazing.* (GP27/F/5
11	Prakash kumaraswamy et-al 2009	Reviewed 241 case notes for patients referred under the two-week wait system with suspected testicular tumour during a complete 3-year period (2003–2005) and recorded information from the referral letter.	The objective was to evaluate the two-week wait referral system for suspected testicular cancer and to compare waiting times from referral to treatment before and after the introduction of the two-week wait process.	For 37 patients, there was a history of a previous vasectomy; usually this was noted in the urology clinic and not in the GP referral letter. In 17 of these cases (46%), the referred swelling was thought to relate to the previous vasectomy. However, a previous vasectomy did not avoid a risk of tumour and three were subsequently diagnosed in this group (1 seminoma, 1 lymphoma and 1 benign Leydig cell tumour).<br><br>The two authors who reviewed the referral letters judged that, in 115	1) The impression that GPs in Cornwall were referring too many older men for suspected testicular cancer is correct in comparison to UK data. From the 241 cases referred under the two-week wait rule in this study, 30% of men were > 55 years of age. After carefully examining the notes and referral letter for evidence of a previous vasectomy. It is clear that the referring GP rarely mentioned such a history when this was identified by the urologist and it was obvious that hardly any

				patients (48%), the referral did not fit the guidelines. In 43 instances (18%), judged that there was a flagrant breach of protocol. In two cases, the referral letter suggested that the GP had not actually examined the patient. Overall, 158 (66%) of the two-week wait referrals underwent an ultrasound scan of the testes and every tumour found in the series had ultrasound confirmation before surgery. Although GPs had referred 241 patients as suspected of having a testicular tumour, a diagnosis of testicular tumour was only made on initial examination in 29 cases by an urologist and a total of 23 tumours were subsequently confirmed.<br><br>Therefore, the final yield of tumours was only 10% and for malignant tumours was just 8% of two-week wait referrals. It is important to note that not a single tumour was found from an inappropriate referral. Two testicular tumours were identified in patients thought to have a benign epididymal swelling on initial examination by a urologist; one was a benign adenomatoid tumour of the epididymis and the second was a spermatocytic seminoma in a 74-year-old patient.<br><br>The majority of patients (56%) were re-assured and discharged with no treatment after initial assessment. In total, 187 patients (78%) did not require any surgical intervention at all and only 28 (12%) went on to have elective surgery for hydroceles and epididymal cysts, etc. Overall, 74 cases (31%) received treatment of some kind following referral, including antibiotics. When the pathology laboratory database was compared with this list of two-week wait referrals only two other tumour case were found one of which had been referred to the private sector and the other via a traditional letter.	GPs appreciated that vasectomy can cause swelling of the epididymis or other masses such as a palpable vasectomy site or sperm granuloma.<br><br>2) It was also shown that vasectomy patients can develop a testicular tumour but, nonetheless, the frequent referral of worried men with no abnormality other than the postoperative features from a vasectomy might merit some discussion in guidelines. The principal findings of this study are that GPs are very poor at examination of the scrotum and weak at interpreting their own findings. The nature of many of the referred swellings was easily determined using a pen torch and there was scant reference to trans-illumination in the referral letters. From the 241 referrals from GPs of suspected testicular tumour, only 29 tumours were suspected on clinical examination by a urologist, only 23 (10%) tumours confirmed by ultrasound and 20 (8%) of these were malignant.<br><br>3) When comparing the GP's description of the examination with the guidelines, 48% of the referrals were judged not to fit the criteria and 18% flagrantly broke protocol. This study clearly show that GPs should strictly abide by the referral criteria and that to do so would dramatically reduce the burden from these patients in urology clinics. **(Adherence to guidelines)**
12	Maria Theresa et-al 2015	Qualitative research using semi structured interviews with GP practitioners, oncologist and colorectal surgeons	To ascertain the challenges associated with implementation of the 2-week wait referral criteria and waiting time targets for colorectal cancer and to identify recommendations for improvements to the pathway.	**Challenges applying the referral criteria for TWW referral** Patients don't always present with those red flags. All groups highlighted the problems applying the TWW referral criteria for colorectal cancer (CRC) to individual patients. GPs reported difficulty applying the referral guidelines, particularly when patients presented with non-specific symptoms, and for those with comorbidities. Specialists acknowledged the difficulty of applying the guidelines and noted that GPs encounter large numbers of patients with non-specific but potentially cancerous symptoms, out of whom only a very small proportion will eventually be diagnosed with cancer:<br>**To refer or not to refer:** In all three geographical areas, there was reported variation in referral practices. Some GPs were happy to refer patients under the TWW pathway about whom they had concerns, but who did not strictly meet the TWW criteria sometimes with the complicit agreement of secondary care specialists. Others felt unable to do so despite having serious concerns that a patient might have a cancer:<br>GPs also raised the issue of having to refer patients who fulfilled the criteria but for whom the probability of cancer was low: non-referral was deemed negligent, but the practice undermined professional judgement and was a poor use of resources.	Quotes 1 ['' *[[diagnosis is difficult for] people who may come with very vague symptoms. People who may have had a diagnosis of a previous bowel problems…Patients with other multimobidity, comorbidities so it's difficult to decide whether it's related to a potential new diagnosis or it's already related to some other factor of that…sometimes patients don't always present with those red flags…you have difficulty getting that patient seen within 2 weeks because they don't fit the criteria.*<br>*[For colorectal cancer GI symptoms are very common within the population…and if you think about the GP they may see less than 1 patient a year with bowel cancer so for them it's hard to pick out the cancer from the non-cancer.*<br>Quote 2 *[The dilemma is if you have somebody who you think it's not quite right but I can't quite tick all the boxes on my 2 wait week form and I don't want to use a 2 week wait system if it's not appropriate because that means somebody else with cancer potentially might have to wait longer.]*<br>*[I know the consultants well enough to be able to phone them up and say it's out of the the 2 week rule guidelines but they really need to be seen and I've done that several times and the consultants have seen them. Or they've said just stick them on the 2 week rule pathway*
13	P. R. Brocklehurst, 2009	Semi-structured interviews were undertaken with eighteen dentists in Sheffield, transcribed and analysed using thematic analysis.	The aim of this study was to use qualitative methods to understand in more detail how practitioners manage potentially malignant lesions once they have been discovered.	**Mechanism of referrals and feedbacks**<br>Real concern about a lesion for many dentists would prompt immediate action to contact secondary care directly. Some PCDs would also maintain contact with the patient if they are concerned. A number of factors that can have an impact on the detail of the referral	*10.116 '…but I would tend to…you know…if I am really concerned…send the patient down…'*<br>*74.161 '…and if I then went outside and found say one or two obvious lymph nodes that were really swollen as well…then I would be going…you know ring up the Charles Clifford and*

				process were also cited by the participants, including the PCDs' confidence and their relationship with the referring hospital. For many, the locality of the dental hospital was a significant factor for the smooth running of the referral service.	*say I want you to see someone today…'*<br>*10.380 '…if it was something that I was very concerned about then I would… I would…on the odd occasion…then I would…you know…keep in touch with the patient…' 66.249 '…patients are also told that if they haven't heard from the dental hospital to contact me… …because we need to have a safety measure in case…'*<br>*40.164 '…if I write it down then I'm confident…if I am not confident then I don't put anything…try and as many of the symptoms and signs that I can but… reluctant to pin my colours to the mast… and state exactly what I think it is…*<br>*77.282 '…because we are in Sheffield… we have good access to the hospital…they know us…so…we'll ring them up…'45.198 '…we are so near the dental hospital that I actually know the clinic…who is working in there…who to send it to…who to address it to…*

.
